# Chemical composition, antioxidant activity and antibacterial mechanism of action from *Marsilea minuta* leaf hexane: methanol extract

**DOI:** 10.1186/s13065-018-0476-4

**Published:** 2018-10-20

**Authors:** Selvaraj Arokiyaraj, Rajaraman Bharanidharan, Paul Agastian, Hakdong Shin

**Affiliations:** 1 Department of Food Science and Biotechnology, College of Life Science, Sejong University, Seoul, 05006 Republic of Korea; 2 Department of International Agricultural Technology, Graduate School of International Agricultural Technology, Seoul National University, Pyeongchang, Gangwon 25354 Republic of Korea; 3 Institute of Green Bioscience and Technology, Seoul National University, Pyeongchang, Gangwon 25354 Republic of Korea; 4 Department of Plant Biology and Biotechnology, Loyola College, Nungambakkam, Chennai, 600034 India

**Keywords:** *Marsilea minuta*, Leaf extract, Antioxidant, Natural preservative, Docking analysis

## Abstract

**Background:**

In the present study, hexane: methanol (50:50) leaf extract of *Marisela minuta* has been evaluated for its chemical composition, antioxidant effect and the antimicrobial mechanism of action against food borne pathogenic bacteria.

**Results:**

The phytochemical evaluation of extract by GC/MS revealed the major abundance of benzoic acid-4-ethoxyethyl ester (43.39%) and farnesol acetate (18.42%). The extract exhibited potential antioxidant and free radical scavenging properties with promising antibacterial activities against the test pathogens with *Pseudomonas aeruginosa* being the most susceptible with maximum inhibition zone (17 mm) and IC_50_ value of 125 µg, respectively. The significant (p < 0.05) increase in intracellular super oxide dismutase (SOD), protein leakage, extracellular alkaline phosphatase and lactate dehydrogenase in treated test pathogens suggested an increase in oxidative stress reveling the mechanism of action of phytochemicals. Scanning electron microscopy analysis of treated pathogens also showed swollen and distorted cells. The bioactive molecules in the extract were efficiently docked with virulent enzymes and farnesol acetate showed best energy value of − 5.19 and − 4.27 kcal/mol towards Topoisomerase IV and SHV-2 respectively. Benzoic acid-4-ethoxyethyl ester showed best binding against TEM-72 with low binding energy value of − 4.35 kcal/mol.

**Conclusion:**

Due to its antioxidant and antibacterial properties, the leaf extract of *M. minuta* may act as promising natural additives to prevent food spoilage bacteria.

## Introduction

The rise in prevalence of multi-drug resistant bacteria has been accredited to undiscriminating use of broad-spectrum antibiotics [1–3]. Nowadays increase of emerging antibiotic resistant bacteria has become a worldwide concern. These drug resistant organisms also can contribute to the risk of food contamination. There have been reports for some drug resistant bacteria like *Pseudomonas aeruginosa, Staphylococcus aureus* and *Enterococcus faecalis* as potent food contaminants [4, 5]. The addition of preservatives has been an effective method to control microbial contamination and authorised synthetic preservatives are still being used to prevent microbial spoilage of processed food. Recently, there is an increasing customer awareness regarding to chemical preservatives in processed food. Considering the demand for natural products with high safety and biological properties, plant compounds has attracted the attention of researchers globally.

Plant secondary metabolites like flavonoids and other phenolic compounds are widely occurring phytochemicals reported to possess antioxidant and antimicrobial properties [6–8]. Many research studies reported plant secondary metabolites exhibit good antioxidant properties [9, 10] and the metabolites from plant origin have a wide spectrum of antimicrobial action against food-borne pathogens and spoilage bacteria [11]. Therefore, the pharmaceutical industries are still in the search of active drug molecules from the unexploited medicinal plants, which exhibit good biological effects (antioxidant and preservative). In plant extracts, massive amount of constituents are present but not all of those are related to pharmaceutical applications. By using chromatography techniques, these phytochemical constituents can be identified, sub-fractionated and tested for their biological properties and many studies reported the chemical composition from plant extracts using GC–MS analysis [12, 13].

In silico studies are preliminary approach to screening novel drug candidates and an emerging strategy to reduce many complexities of drug discovery process and this method has played important role in the rational drug design to identify the biological or phytocompounds potential against antimicrobial resistant proteins [14]. In the present study, we selected *Marsilea minuta* Linn (Marsileaceae) leaves material for exploring its biological potential. *M. minuta* commonly found in the banks of ponds and canals and as a weed in the wet rice fields and distributed throughout India. It has a great traditional medicinal value possessing anti-infertility [15], anti-depressant [16], hypocholesterolemic [17] and hepatoprotective activities [18]. Earlier studies investigated the antibacterial activity of gold nanoparticles synthesized from the *M. minuta* leaf extract against *Escherichia coli* and *Staphylococcus aureus* [19] and antibacterial activity against various pathogens have also been reported [20]. However, there are no reports on the complete phytochemical composition and the mode of action of extracts from *M. minuta* against food borne pathogens. Therefore, the objective of this work is to evaluate the chemical composition, antioxidant activity, antimicrobial activity, and the mode of action against food borne pathogens of *M. minuta* leaf extract.

## Results and discussion

### Chemical composition of the *M. minuta* leaves extract

GC–MS analysis of *M. minuta leaves* extract (50% hexane:50% methanol) identified 12 compounds and the predicted constituents in the extracts were listed in Table 1. The major compounds were benzoic acid-4-ethoxy-, ethyl ester (43.39%), a monoester of benzoic acid and farnesol acetate (18.42%), a sesquiterpene compound. These two chemical molecules selected for molecular docking studies with target proteins TEM-72 and Topoisomerase IV for their possible antibacterial mechanism of action. Earlier studies reported that farnesol was potentially active against *Staphylococcus aureus* and *Streptococcus mutans* [21, 22] and benzoic acid-4-ethoxy-, ethyl ester used in stabilizers in preparation of packaging material [23]. Next, phenol, 2,4-bis (1,1-dimethylethyl) (8.37%), a phenolic compound; oxacycloheptadec-8-en-2-one (5.68%), a lactone; and trans-farnesol (5.11%), an oxygenated sesquiterpene were identified. The presence of phenolic compounds may possess antioxidant and antibacterial mechanism and there are numerous reports available on phenolic compounds exhibiting antioxidant, antimicrobial, heptaprotective and antidiabetic potential [24, 25]. Therefore, the chemical constituents found in *M. minuta leaves* extracts may play major roles in the antioxidant and antimicrobial properties.

**Table 1:** GC–MS analysis of *Marsilea minuta* leaves extract

Peak no	Components	Class of compound	Retention time	Area %
1.	Phenol, 2,4-bis (1,1-dimethylethyl)	Phenol	13.52	8.37
2.	Benzoic acid, 4-ethoxy-, ethyl ester	Aromatic acid ester	13.76	43.39
3.	1,6,10-dodecatrien-3-ol,3,7,11-trimethyl	Oxygenated sesquiterpene	14.20	2.61
4.	Trans-Farnesol	Oxygenated sesquiterpene	16.00	5.11
5.	2,6,10-Dodecatrien-1-ol,3,7,11-trimethyl-acetate	Sesquiterpene	16.96	1.71
6.	Farnesol, acetate	Sesquiterpene	17.22	18.42
7.	Phthalic acid, isobutyl undecyl ester	Diester	17.54	4.91
8.	7,9-Di-tert-butyl-1-oxaspiro(4,5)deca-6,9-diene-2,8-dione	Spirolactone	18.05	1.84
9.	Oxacycloheptadec-8-en-2-one	Lactone	18.46	5.68
10.	1,2-Benzenedicarboxylic acid, butyl-2-methylpropylester	Diester	18.51	4.84
11.	Octadec-9-enoic acid	Unsaturated fatty acid	19.64	1.89
12.	Oleic acid	Unsaturated fatty acid	21.23	1.22

Compound proportions were calculated from the chromatograms obtained on the TG-5MS column. The percentage of the compounds detected in the GC that was calculated based on the relative area of individual compounds to the total area of the components identified from the extract

### Ferric reducing antioxidant power assay

**Fig. 1:**
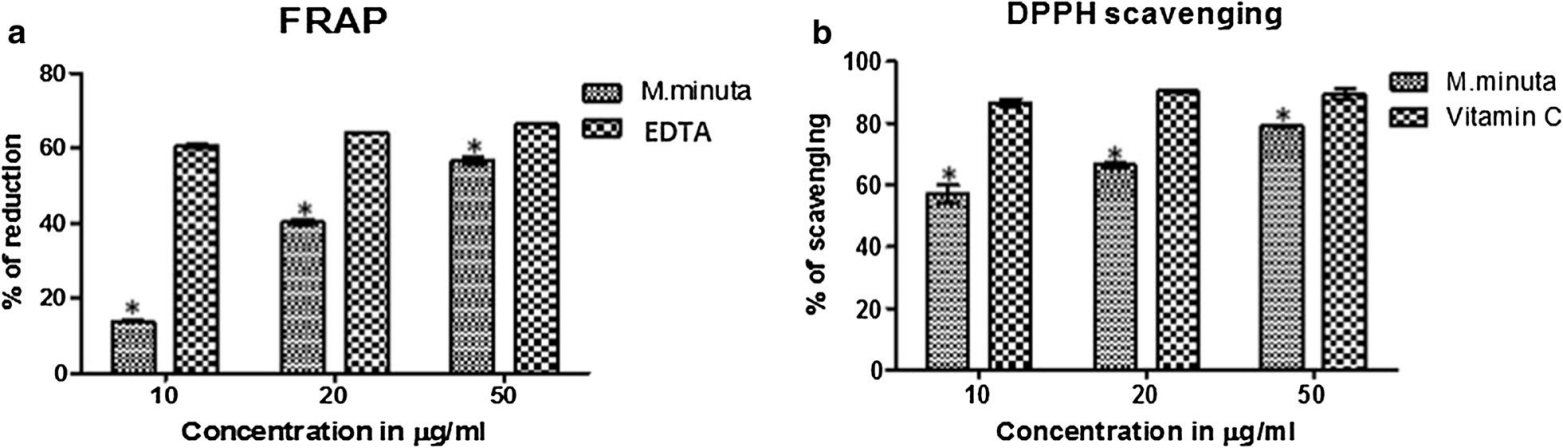
**a** FRAP scavenging activity of *M. minuta* leaves extract (%), **b** percentage inhibition of DPPH free radical by *M. minuta* leaves extract. Values represent the mean ± SEM of triplicate, independent experiments; the values labeled with Asterisk indicate statistically significant difference compared with standard compound as determined by Student t-test (p < 0.05)

The *M. minuta* extract showed a significant dose-dependent inhibition of FRAP activity. The highest reducing activity (60%) found in the concentration of 50 µg/ml when compared with the standard EDTA (Fig. 1a). The IC_50_ concentrations for the standard and *M. minuta* leaves extracts were found to be 7.42 and 37.48 µg/ml (Table 2) respectively. The reducing ability effect of *M. minuta* extracts was mainly due the presence of phytochemical compounds. Also, the presence of phenolic compounds can contribute the reduction potential. In general, the antioxidant activity of phenolic compounds is due to their ability to chelate metal ions involved in the generation of free radicals [26]. In support of the antioxidant effect, GC–MS spectrum confirmed the presence of phenolic compounds.

**Table 2:** IC50 value of FRAP and DPPH radical scavenging activity

Antioxidant activity	*M. minuta*	EDTA	Vitamin C
FRAP µg/ml	37.48	7.42	–
DPPH µg/ml	8.94	–	5.77

### Scavenging activity of DPPH radicals

This method depends on the reduction of purple DPPH radicals to a yellow colored diphenyl picryl hydrazine. The reduction of color of DPPH solution indicates an increase of the DPPH radical scavenging activity [27]. The percentage of DPPH scavenging in the presence *M. minuta* leaves extracts at different concentrations were shown in Fig. 1b. The result showed a significant dose-dependent inhibition of DPPH activity and the values were found to be significant (p < 0.05). The IC_50_ concentrations for the standard (vitamin C) and *M. minuta* extracts were found to be 5.77 and 8.94 µg/ml, respectively. The extract exhibited concentration dependent activity and the presence of certain phytochemicals may result in the free radical scavenging potential. Moreover, our results are in agreement with previous findings demonstrating DPPH scavenging effect of methanolic extract of *M. quadrifolia* [28].

### Antibacterial activity

In this study, we tested antibacterial ability of *M. minuta* leaves extract against *Bacillus subtilis*, *Staphylococcus aureus*, *Enterococcus faecalis*, *Klebsiella pneumonia* and *Pseudomonas aeruginosa.* These bacteria are associated with food borne diseases, food spoilage and multi drug resistant bacteria [29]. Antibacterial assay results showed *M. minuta* leaves extract exhibited good inhibitory effect against all of the test strains (Table 3). Among the tested pathogens *P. aeruginosa* exhibited the maximum inhibition zone (17 mm). Our results are in accordance with Gupta et al. [30], that the ethanolic extract of *Achyranthes aspera*, *Cynodon dacynodon dactylon*, *Lantana camara* and *Tagtes patula* showed effective antibacterial activity against *S. aureus, P. aeruginosa and B. subtilis*. Likewise, the minimum inhibitory concentration (MIC) of the *M. minuta* leaves extract against the tested strains of various bacterial pathogens with concentration ranging from 125 to 250 μg/ml (Table 4). Our results are in agreement with the reports of Rios and Recio [31], that plant extract possessing an MIC value equaling or less than 1000 μg/ml is considered to be active and worthy antimicrobials. In the present study, *M. minuta* leaves extract possesses a variety of phytochemicals. Therefore, the antibacterial activities of *M. minuta* may be due to the presence of phenolic compounds (phenol-2,4-Bis(1,1-dimethylethyl)) as well as different concentration of aromatic acid ester, oxygenated sesquiterpene, sesquiterpene and fatty acids [32]. Similarly, Prakash and Suneetha [33] reported the presence of phenolic compound (phenol-2,4-Bis(1,1-dimethylethyl)) in the *Pinus granatum* extract and showed potential antioxidant activity. The probable mode of antibacterial action may be due to disruption in cell membrane, lysis and leakage of intracellular compounds [34]. However, because of the heterogeneous compositions of the *M. minuta* leaves extracts, the individual compounds responsible for its antimicrobial mechanism need to be identified.

**Table 3:** Antibacterial activity (zone of inhibition, mm) of *M. minuta* leaves extract

Bacterial species	*M. minuta*	Streptomycin
*E. faecalis*	16 ± 0.42	26 ± 0.72
*B. subtilis*	16 ± 0.38	24 ± 0.45
*P. aeruginosa*	17 ± 0.27	25 ± 0.33
*K. pneumonia*	12 ± 0.34	26 ± 0.76

Values are mean of experiments performed in triplicate and data are expressed as mean ± SD

**Table 4:** Minimum inhibitory concentration of *M. minuta* leaves extract

Species	MICs (µg/ml)
*E. faecalis*	250
*B. subtilis*	250
*P. aeruginosa*	125
*K. pneumonia*	250

### SOD quantification

**Fig. 2:**
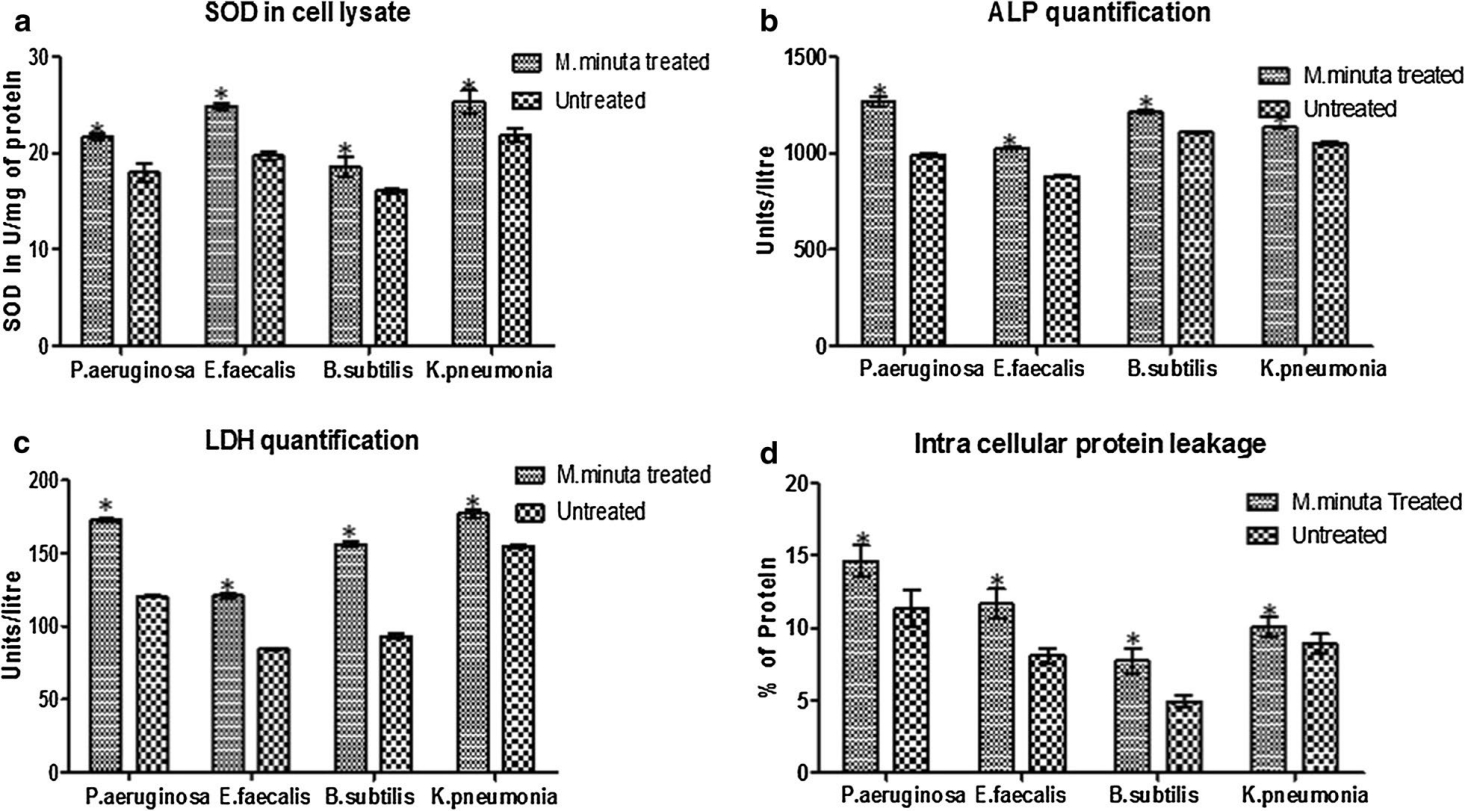
**a** Quantification of SOD level in *M. minuta* leaves extract treated bacterial species; **b** quantification of ALP level in *M. minuta* leaves extract treated bacterial species; **c** quantification of LDH level in *M. minuta* leaves extract treated bacterial species; **d** assessment of intracellular protein leakage of bacterial species treated with *M. minuta* leaves extract. Values represent the mean ± SEM of triplicate, independent experiments; the values labeled with Asterisk indicate statistically significant difference compared with untreated bacteria as determined by Student t-test (p < 0.05)

Superoxide dismutase (SOD) enzymes present in aerobic and anaerobic organisms responsible for the breakdown of superoxide radicals [35]. When SOD activity was high, it leads to the increase in tolerance to oxidative stress; secondly, increased stress leads to cell wall damage and cell burst. Similarly, in our study, we observed SOD quantity for all the treated bacteria was high when compared with untreated bacteria and the values were significant (p < 0.05). The results for the quantification of SOD levels in *M. minuta* leaves extract treated and untreated bacteria are shown in Fig. 2a. This clearly shows that the extract exhibited a stress towards the pathogens. Similarly, Dwyer et al. [36] reported that treatment of *Escherichia coli* with bactericidal antibiotics induced the generation of ROS, via a common metabolic mechanism, which contributes to drug-induced killing.

### ALP quantification assay

In bacteria, alkaline phosphatase (ALP) is usually located in the periplasmic space to generate free phosphate groups for uptake and use. More amount of alkaline phosphatase is usually produced during phosphate starvation and sporulation. In the present study, we observed significant increase (p < 0.05) in the ALP level in the bacteria treated with *M. minuta* leaves extract (Fig. 2b). The increase may be because of stress imposed on the bacteria by the extract, and in order to overcome the starvation, the bacteria produces more amount of ALP. Previous studies revealed that the ALP levels were increased in *Clostridium perfringens* and *Brachyspira hyodysenteriae* upon treatment with Quinoxaline 1,4-di-*N*-oxide derivatives compared to the non-treated groups [37]. Therefore, the observed significant increase in the ALP activities in the bacteria treated with *M. minuta* extract suggests an increase in the activities of the existing enzymes by the secondary metabolites.

### LDH quantification assay

The effects of *M. minuta* extract on LDH activities of *S. aureus* and other bacteria were shown in Fig. 2c. The LDH activity in the treated bacterial group were high when compared to the untreated one. The values were significant (p < 0.05) and showed a variance of 120–175 units/l. This indicate that *M. minuta* extract does interact with the bacterial cell surface. *M. minuta*-bacteria interaction mediated by electrostatic forces. After attachment, alternation in membrane permeability causes the leakage of cytosolic enzyme (glucose and LDH), which finally causes cell death [38].

### Intra cellular protein leakage

The *M. minuta* extract was observed to induce protein leakage in all the test organisms (Fig. 2d). Both of the Gram (−) and Gram (+) bacteria showed a similar trend of protein leakage when treated with the *M. minuta* extract. Among all bacteria, *P. aeruginosa* had the highest damaging effect causing leakage compared to untreated bacteria (p < 0.05). This is in agreement with the previous report by Henie et al. [39] indicating measuring protein leakage level could be used as an indicator of membrane damage.

### Scanning electron microscope observation

**Fig. 3:**
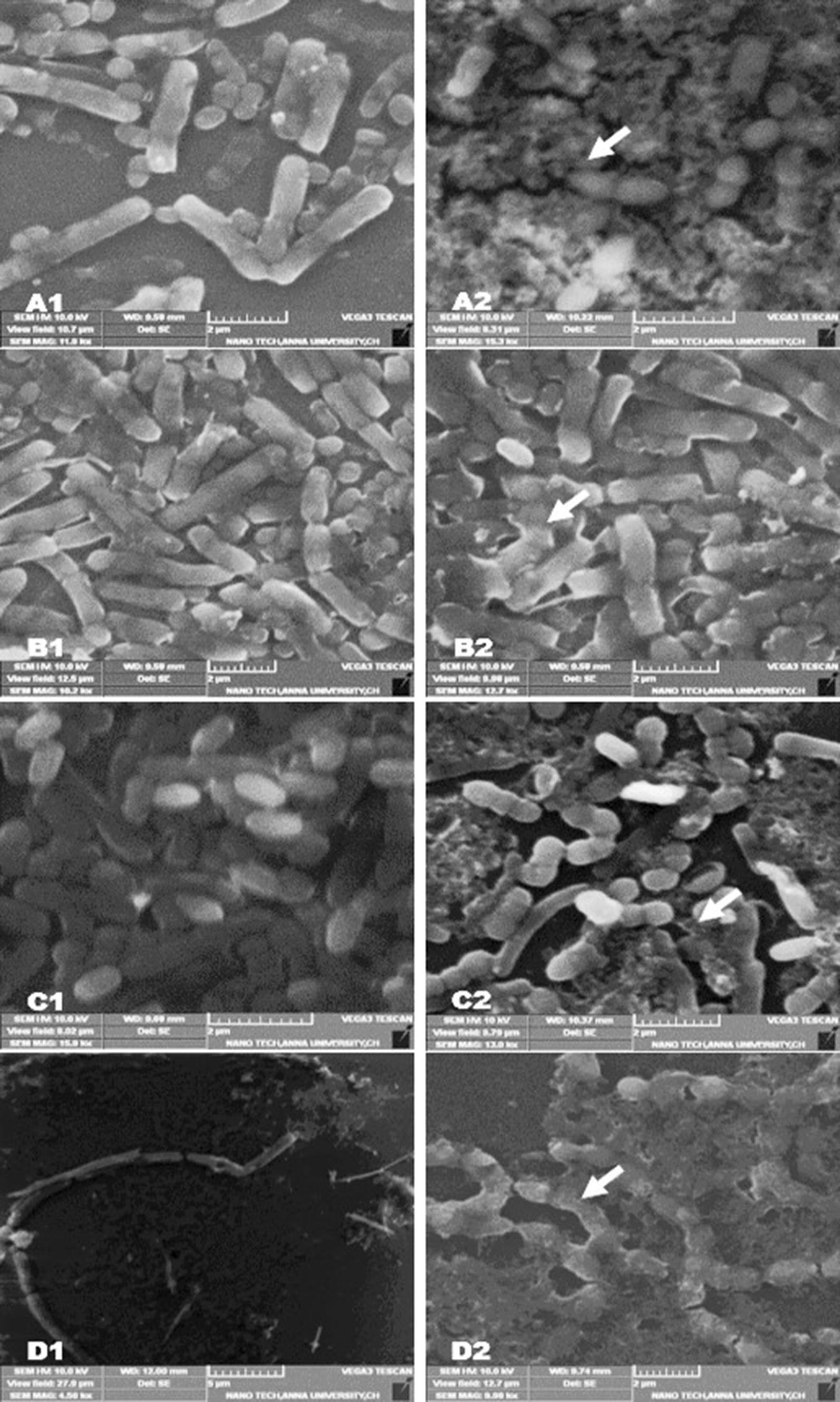
Morphological comparison of bacteria treated with *M. minuta* leaves extract by scanning electron micrograph. Arrows indicates swollen cells, leakage of cell contents and change in cell shape. A1—*Pseudomonas aeruginosa* (Control); A2—*Pseudomonas aeruginosa* (Treatment); B1—*Klebsiella pneumonia* (Control); B2—*Klebsiella pneumonia* (Treatment); C1—*Enterococcus faecalis* (Control); C2—*Enterococcus faecalis* (Treatment); D1—*Bacillus subtilis* (Control); D2—*Bacillus subtilis* (Treatment)

The damage in bacterial cell wall by *M. minuta* extract treatment were extensively studied by scanning electron microscope (Fig. 3). The test bacterial strains *P. aeruginosa, K. pneumonia, E. faecalis* and *B. subtilis* control without *M. minuta* extract treatment showed smooth and damage free cells. Whereas, extract treated bacterial cell showed distortion in their cell morphology causing leakage of intra cellular components and results in cell death. This observation support the conclusion from lactate dehydrogenase and intra cellular protein leakage assay. Similarly, Burt and Reinders [40] observed that oregano and thyme essential oil showed potent antimicrobial properties against *E. coli* and the mode of action to be cell wall degradation; damage in cytoplasmic membrane proteins; leakage of cellular contents, and depletion of proton motive forces.

### Docking study of *M. minuta* ligands with target proteins

**Fig. 4:**
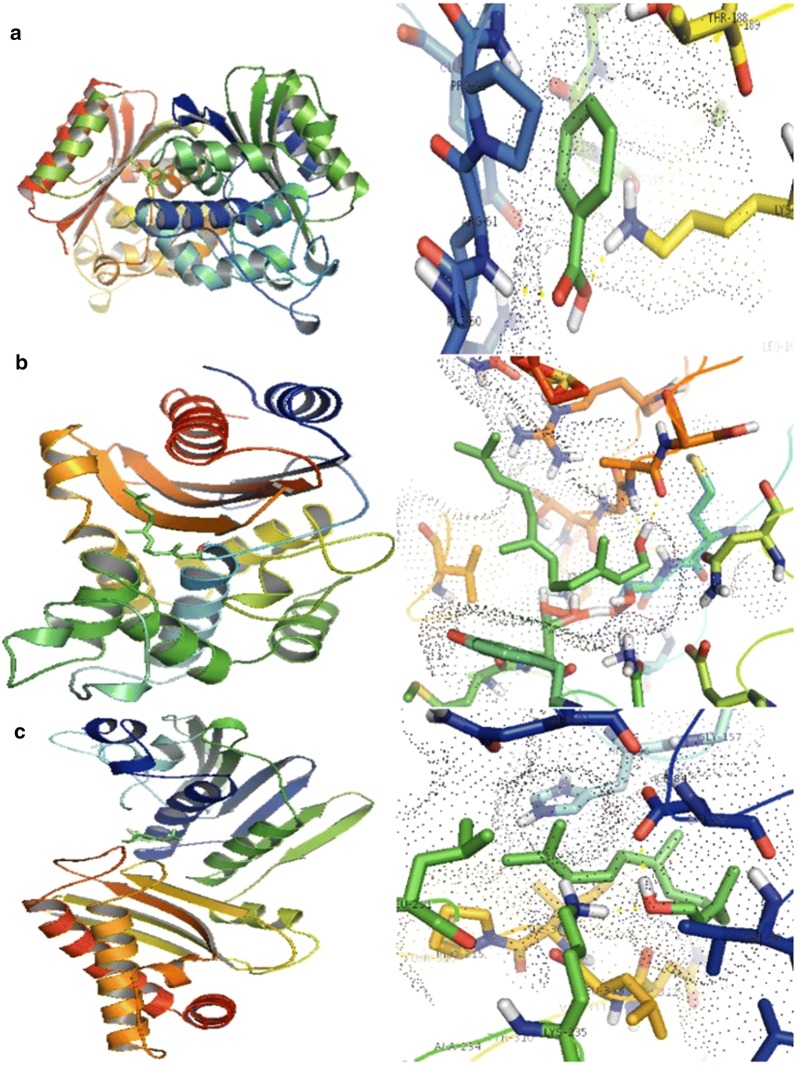
Putative binding poses of ligands docked with TEM-72, SHV-2 and topoisomerase IV. The yellow dotted line indicates the *H*-bonding between the ligand and protein. **a** Molecular interaction of ligand benzoic acid-4-ethoxy-ethyl ester with TEM-72. **b** Molecular interaction of ligand farnesol acetate with SHV-2. **c** Molecular interaction of ligand farnesol acetate with Topoisomerase IV

Bacterial proteins are the ultimate target to inhibit their growth since these are the executors of many cellular functions. Production of extended-spectrum β-lactamases (ESBLs) by bacteria belonging to family *Enterobacteriaceae* is a deep scientific concern, since they are able to neutralize the β-lactam antibiotics making them more resistant to antibiotics. The SHV family of β-lactamases is universally found in *K. pneumoniae* and confers resistance to broad-spectrum penicillins such as ampicillin [41]. TEM-72 a class A, β-lactamases enzyme represent resistant factors against β-lactam antibiotics [42] and topoisomerases help in unwinding the DNA during bacteria replication [43]. Considering these factors TEM-72, SHV 2 and topoisomerases IV were selected for molecular docking studies. After docking studies, we have found that that the ligands (benzoic acid-4-ethoxy-ethyl ester and farnesol acetate) showed satisfactory binding towards the target proteins and the results are shown in Table 5 and Fig. 4. Table 5 represents the energy values of ligand receptor interaction, where farnesol acetate has the best energy value of − 5.91 K Cal/mol towards topoisomerase IV. Lower the energy value, better the ligand docked to the receptor. Hydrogen (H) bonding play a critical role in determining the structure and function of any biological molecule, especially for its inhibition in a complex [44]. The ligand benzoic acid-4-ethoxy-ethyl ester docked complex was stabilized by two H-bond with A:LYS 192 and B:ARG 61 of TEM-72 with lowest binding energy of − 4.35 kcal/mol (Fig. 4a) and another ligand farnesol acetate is stabilized by two H-bonds with residues of A:ALA 237 with lowest binding energy of − 4.27 kcal/mol in SHV-2 (Fig. 4b). This ligand also formed two H-bonds with A:ASP 85 and A:LYS 235 with lowest binding energy of − 5.19 kcal/mol in topoisomerase IV (Fig. 4c). The in silico results showed that, the major compounds (benzoic acid-4-ethoxy-ethyl ester and farnesol acetate) present in *M. minuta* extract having minimum binding energy and have good affinity toward the active pocket, thus, they may be considered as good inhibitor of topoisomerase IV, SHV-2 and TEM-72 protein. Despite from antibacterial and antioxidant activities by *M. minuta* leaves extract, this study has some limitation i.e. we have not conducted bioassay-guided fractionation of bioactive molecules present in the *M. minuta* and the probable mechanism (In silco studies) of action of benzoic acid-4-ethoxy-, ethyl ester and farnesol acetate was based on the major compounds that was predicted by GC–MS analysis. In addition, the extract may have non-volatile bioactive compounds in addition to the reported compounds. Therefore, detailed analysis of the total chemical constituents of this plant and bioassay guided fraction of bioactive metabolite will be conducted in future studies.

**Table 5:** The docking scores of the ligands with the target protein

Protein	Ligand	Binding energy (kcal/mol)
TEM-72 (PDB ID: 3P98)	Benzoic acid-4-ethoxy-ethyl ester	− 4.35
SHV-2 (PDB ID: 1N9B)	Farnesol acetate	− 4.27
Topoisomerase IV (PDB ID: 3LPS)	Farnesol acetate	− 5.19

## Experimental details

### Chemical reagents and solvents

Folin-Ciocalteu reagent, 2,2-diphenyl-1-picrylhydrazyl (DPPH), Sodium carbonate, Aluminum chloride, *O*-phenanthroline, EDTA, Nitro Blue Tetrazolium dye (NBT), NaOH, *p*-nitrophenol, CaCl_2_,Trichloroacetic acid (TCA) and n-hexane, methanol were purchased from Sigma Chemical Co., Ltd (St. Louis, MO,USA). All other chemicals and solvents used were of analytical grade (AR) and purchased from Himedia, India.

### Microorganisms

*Bacillus subtilis* (ATCC 9372), *Enterococcus faecalis* (ATCC 29212), *Klebsiella pneumoniae* (ATCC 9621), *Pseudomonas aeruginosa* (ATCC 27853) and *Staphylococcus aureus* (ATCC 25923) were obtained from the Pondicherry center for biological sciences (PCBS), Pondicherry, India. All bacterial cultures were maintained in Mueller–Hinton Agar (MHA, Himedia, India) slants and stored at − 20 °C.

### Plant collection and extract preparation

Fresh leaves of *M. minuta* were collected from the region of Gopalapuram, Cuddalore district, Tamil Nadu, India. A botanist authenticated the leaves specimen and the voucher specimen deposited in the laboratory. The leaves of *M. minuta* were shade dried (10 days) and powered by using grinder. For extraction, we have first extracted the sample-using methanol. Further, the methanol solution re-extracted by liquid–liquid extraction using hexane: methanol (50:50 v/v) ratio. The later liquid–liquid extraction was conducted to remove the fat content in the methanol extract [45]. The extract yield (pale brownish in color) was 17.84% (v/v). The extracts were dehydrated over anhydrous sodium sulfate and stored at 4 °C in air-tight glass vials until use.

### GC–MS analysis

The *M. minuta* hexane: methanol extract was analysed by a Thermo Trace 1310 (Gas chromatograph) system, fitted with a TG-5MS (Mass spectroscopy) column (30 × 0.25 mm (5%-phenyl)–methylpolysiloxane capillary column, coating thickness × 0.25 µm), 220 °C temperature injector and 250 °C temperature transfer line. The oven temperature was held at 50 °C for 5 min, and then programmed to 250 °C at rate of 4 °C/min. The ionizing energy was 70 eV. The amount of sample injected was 1 µl (split ratio 1:10). Identification of unknown components in *M. minuta* extracts were determined by comparing the retention times of chromatographic peaks using Quadra pole detector with the National Institute Standard and Technology (NIST MS search Program V.2.0 g) library to relative retention indices. Quantitative determinations were made by relating respective peak areas to total ion chromatogram areas from the GC–MS [46].

### Ferric reducing antioxidant power (FRAP) assay

The FRAP activity was determined by colorimetric method [47]. The reaction mixture containing 1 ml of 0.05% *O*-Phenanthroline in methanol, 2 ml ferric chloride (200 μM) and 2 ml of various concentrations (10 to 50 μg) of *M. minuta* extracts, incubated at room temperature for 10 min and the absorbance of the sample was measured at 510 nm. Moreover, the IC_50_ value was calculated. The experiments were performed in triplicate.

### DPPH free radical scavenging assay

DPPH radical scavenging capacity and quenching ability of *M. minuta* leaf extract were estimated by following the methods reported by Zhang and Hamauzu [48]. Hexane: methanol extracts with different concentration (10–50 μg/ml) were mixed with DPPH solution (0.15%) in methanol. Then it was incubated at dark for 10 min and the absorbance was read at 517 nm. The antiradical activity was expressed as IC_50_ (μg/ml), (the antiradical dose required to cause a 50% inhibition). Vitamin C was used as standard. The percentage inhibition was calculated using the following formula:

$$\% {\text{ Scavenging}} = \left[ {\left( {{\text{A}}_{\text{o}} - {\text{A}}_{\text{s}} } \right)/{\text{A}}_{\text{o}} } \right]* 100$$

where, A_o_ is absorption of control, and A_s_ is absorbance of sample and standards respectively. Moreover, the IC_50_ value was calculated [47]. The experiments were performed in triplicate. For both FRAP and DPPH assay, the reagent and buffer, free of the plant extract was used as control. All colorimetric assays were performed using ALERE microplate reader (Alere Medical Pvt Ltd, India, AM 2100).

### Superoxide dismutase (SOD) quantification

SOD activity was done based on the reduction of superoxide-nitroblue tetrazolium complex according to a previously reported protocol [49]. The assay mixture contained 25 µl cell supernatant (microbial cell) obtained by lysing the extract treated cells by Triton X-100, with 0.05 ml of l-methionine (200 mM), and 0.05 ml of nitro blue tetrazolium (1.5 mM NBT) solution. The enzyme activity was measured by measuring the reduction of NBT with xanthine oxidase as a hydrogen peroxide generating agent. The reaction mixture was illuminated for 30 min and the absorbance at 560 nm was measured against the control and test samples.

### Alkaline phosphatase (ALP) quantification

Bacteria were cultured in MHB treated with 1 mg/ml of *M. minuta* leaf extract. After 14 h of incubation, cell free supernatants were collected for ALP assay. The assay was performed using ALP assay kit (Linear Chemicals, Montgat, Barcelona, Spain) by following the procedure as reported earlier [50]. To measure the ALP activity, extract treated samples were compared with control (cells without treatment) and the results were expressed in units/liter.

### Assessment of antibacterial activity

The antibacterial activity of *M. minuta* leaves extract was performed by well diffusion method. Respective bacterial cultures were swabbed onto sterile petri plates containing Muller Hinton agar using sterile cotton swab. Then wells of 6 mm in diameter were made and 30 µl (30 μg) of extracts and 30 µl of streptomycin (30 μg/ml; used as positive control) were added to each well. Further, the plates were incubated at 37 °C for 14 h. After incubation, the antibacterial activity was measured in terms of zone of inhibition (mm). The experiments were performed in triplicate.

### Minimum inhibitory concentration (MIC)

A twofold serial dilution of *M. minuta* extracts in Mueller–Hinton broth had been prepared in 96-well micro titre plate [51]. A standardized inoculum for each bacterial strain (10^6^ CFU/ml) was prepared in each well. Streptomycin was used as a control. The plate was kept at 37 °C and incubated for 14 h. MIC was calculated as the lowest concentration of the extracts inhibiting the visual growth of the test cultures on the agar plate.

### Lactate dehydrogenase (LDH) quantification

The presence of the cytosolic enzyme (LDH) in the cell culture medium is the indicative of cell membrane damage. The LDH activity was determined by measuring the reduction of NAD^+^ to NADH and H^+^ during the oxidation of lactate to pyruvate. The activity was measured using LDH cytotoxicity assay kit (Linear chemicals, Spain), in accordance with manufacturer’s instructions. The percent of LDH released from the cells was determined using the units/L of protein.

### Intracellular protein leakage

The bacterial cultures were treated with 1 mg/ml of *M. minuta* leaf extract and incubated for 14 h at 37 °C. After incubation, the cells were centrifuged at 5000 rpm for 10 min and the supernatants were collected. To determine the intracellular protein leakage, the supernatant was assayed according to the method of Bradford M.M [52].

### Scanning electron microscope observation (FE-SEM)

The morphological changes of bacterial cells treated with *M. minuta* extracts, were observed under scanning electron microscope (VEGA3 TESCAN) and the procedures were performed according to Kockro et al. [53]. Bacterial cells (10^6^ CFU/ml) were treated with 1000 µg/ml of extracts for 14 h, centrifuged at 3000*g* for 30 min. The pellets were washed three times with phosphate buffered saline and pre-fixed with 10% formaldehyde for 30 min. The pre-fixed cells were washed with 30, 50, 70, 80, 90 and 100% of ethanol.

### In silico molecular docking studies

The major constituents of *M. minuta* leaves extract (hexane: methanol) were subjected to molecular docking studies with three target proteins (TEM-72, PDB ID: 3P98; SHV-2, PDB ID: 1N9B; and Topoisomerase IV, PDB ID: 3LPS). Search of protein data bank confirmed presence of 3D structures of ESBL TEM-72 (at 2.10°A resolution), SHV-2 (at 0.90° A resolution) and Topoisomerase IV (at 0.98°A resolution) proteins. To analyze the nature of interactions with bioactive compounds, docking was carried out using AUTODOCK 4.0 and other docking procedures were followed as reported in our earlier work [50].All figures with structural representation were produced using PyMol [54].

### Statistical analysis

The results obtained from cultured cells were analysed by Student’s *t* test. Statistical analysis were carried out using statistical package for the social sciences software (SPSS version 21; SPSS Inc., Chicago, USA) and p < 0.05 were considered as significant.

## Conclusion

In the present study, the results indicated that *M. minuta* leaves extract showed antioxidant, antibacterial effect against food pathogens by disrupting their outer membrane and in silico docking analysis showed the major compound (benzoic acid-4-ethoxy-ethyl ester and farnesol acetate) exhibited good affinity towards of topoisomerase IV, SHV-2 and TEM-72. These results suggest that *M. minuta* may act as promising natural additives to prevent food spoilage bacteria. Moreover, the present study is a preliminary experiment to screen bioactive metabolite profile of *M. minuta* leaves and here we have used the GC/MS analysis as a tool to report the chemical constituents. Therefore, further studies are needed to validate the novel antibacterial bioactive molecules.
